# Gelatin-Modified Polyurethanes for Soft Tissue Scaffold

**DOI:** 10.1155/2013/450132

**Published:** 2013-11-20

**Authors:** Justyna Kucińska-Lipka, Iga Gubańska, Helena Janik

**Affiliations:** Department of Polymer Technology, Chemical Faculty, Gdansk University of Technology, Narutowicza Street 11/12, 80-233 Gdansk, Poland

## Abstract

Recently, in the field of biomaterials for soft tissue scaffolds, the interest of their modification with natural polymersis growing. Synthetic polymers are often tough, and many of them do not possess fine biocompatibility. On the other hand, natural polymers are biocompatible but weak when used alone. The combination of natural and synthetic polymers gives the suitable properties for tissue engineering requirements. In our study, we modified gelatin synthetic polyurethanes prepared from polyester poly(ethylene-butylene adipate) (PEBA), aliphatic 1,6-hexamethylene diisocyanate (HDI), and two different chain extenders 1,4-butanediol (BDO) or 1-ethoxy-2-(2-hydroxyethoxy)ethanol (EHEE). From a chemical point of view, we replaced expensive components for building PU, such as 2,6-diisocyanato methyl caproate (LDI) and 1,4-diisocyanatobutane (BDI), with cost-effective HDI. The gelatin was added in situ (in the first step of synthesis) to polyurethane to increase biocompatibility and biodegradability of the obtained material. It appeared that the obtained gelatin-modified PU foams, in which chain extender was BDO, had enhanced interactions with media and their hydrolytic degradation profile was also improved for tissue engineering application. Furthermore, the gelatin introduction had positive impact on gelatin-modified PU foams by increasing their hemocompatibility.

## 1. Introduction

Cellular scaffold is defined as extracellular matrix (ECM), which surrounds cells in the body. Its main task is physical support and regulation of cells proliferation. In addition, ECM supports cells to merge and moreover affects their shape and movement and also direct functions [1]. A vast number of cellular scaffolds were obtained from natural polymers (chitosan, elastin, alginate, and collagen) and synthetic ones (polyglycolide, polylactide, polyurethane) or even ceramic materials (hydroxyapatite and bioglass) [2–6]. Synthetic scaffold, implanted in the affected area, after fulfilling its task (cells growth) should degrade [7–11]. One of synthetic polymers applicable as cellular scaffolds is medical grade polyurethane (PU), which does not interact with body fluids or cause blood clotting. In addition, its physicochemical properties can be easily modified, because of its segmented structure, which can be regulated by the type of used substrates in the synthesis [7]. Polyurethanes degrade slower than the other synthetic polymers (poly (lactic acid) (PLA), polyglycolide (PGA)), and they may be especially used as hard tissue implants [12–14]. Materials for soft tissue scaffolds should undergo more rapid degradation than the materials for hard tissue scaffolds. Therefore, polyurethanes are widely modified with natural polymers and one of the commonly used natural polymers is gelatin (collagen derivative), which is biocompatible material for medical devices, approved by the Food and Drug Administration (FDA) [15–18]. Doi and Matsuda, 1997, used mixed solution of photoreactive gelatin, basic fibroblast factor (bFGF), and heparin to obtain coated microporous polyurethanes for artificial vascular grafts with increased porosity and enhanced proliferation of endothelial cells. Three models of segmented polyurethane grafts obtained as tubular films with inner diameter of 1,5 mm, with or without micropores, were fabricated by using an excimer laser ablation technique and their neoarterial regenerative potential was studied upon implantation. The microporous grafts were prepared from segmented polyurethane, called Cardiomat 610. Those tubes were cured by pulsed ultraviolet (UV) light. The implantation study showed that after 4 weeks of implantation the neoarterial tissue regeneration continued thanks to the used gelatin additive [19]. Detta et al., 2010, developed and investigated novel polyurethane-gelatin micro/nanostructure meshes, prepared from commercial elastomeric polyurethane (Tecoflex EG-80A) and gelatin, as blood vessel substitutes (especially for small diameter of vascular prosthesis). Obtained composite meshes had increased mechanical characteristics and showed enhanced endothelial cells adhesion and proliferation [10]. Ulubayram et al., 2001, obtained novel polyurethane bilayer wound dressing containing epidermal growth factor (EGF) loaded in gelatin microspheres. The various porous matrices in sponge form were prepared from gelatin by freeze-drying technique. As the external layer, elastomeric polyurethane membranes were used. The in vivo studies showed that controlled release of EGF from microspheres provided higher degree of wound area reduction. Histological investigations confirmed that the prepared dressings were biocompatible and did not cause any mononuclear cell infiltration or foreign body reaction. The structure of newly formed dermis was almost the same as that of the normal skin [20]. Chong et al., 2007, proposed a novel polyurethane material, modified with gelatin, for artificial “dermal layer,” which adheres and integrates with the wound. The cost-effective composite was prepared in a form of nanofibrous scaffold (PCL/gelatin-type A) directly electrospun onto a polyurethane dressing (Tegaderm, 3 M Medical). Cell studies:fibroblasts seeding-showed that nanofiber construct achieved significant cell adhesion, growth, and proliferation [21]. Kim et al., 2009, prepared nanofiber scaffold using polyurethane (PU) and gelatin (with electrospinning technique) to obtain also a wound dressing material. Studies showed that when the gelatin amount (in the blended solution) decreased, the contact angle increased and the water uptake of the scaffold decreased concurrently. In the mechanical tests, the blended nanofibrous scaffolds were elastic and elasticity increased as the total amount of PU increased. Moreover, as the total amount of gelatin increased, the cell proliferation increased with the same amount of culture time [22]. Guan et al., 2007, described polyurethane-gelatin scaffolds having desirable mechanical properties for cardiovascular purposes. Such scaffolds provide appropriate mechanical environment for tissue reconstruction or healing in vivo. The biodegradable poly(ester urethane)urea (PEUU) scaffolds loaded with bFGF on gelatin were fabricated by thermally induced phase separation. Those scaffolds showed slightly higher degradation rates than unloaded control scaffolds [23]. Sartori et al., 2008, investigated loaded PU systems, by using fibroblasts enclosed in gelatin, in order to control chemistry of materials for promotion of highly specific binding interactions between materials and biological environments. The study showed that such treatment increases cells growth [24]. Adhikari et al., 2010, developed and investigated polyurethane networks containing covalently attached zwitterionic compounds (dihydroxy polycaprolactone phosphorylcholine and 1,2-dihydroxy-N,N-dimethylamino-propane sulfonate), which were mixed with 10% wt of hydrated gelatin beads. Cured gelatin polymer beads showed compression strength suitable for use in articular cartilage restoration [25].

The aim of our study was obtaining novel PU foams that could be used as a soft tissue scaffold, which would enable functional tissue remodeling in place of tissue defect or damage. The aliphatic PU foams were prepared from poly(ethylene-butylene adipate) (PEBA), 1,6-hexamethylene diisocyanate (HDI) and 1,4-butanediol (BDO) or 1-ethoxy-2-(2-hydroxyethoxy)ethanol (EHEE) in two-step polymerization process. In addition, we modified our polyurethanes with gelatin what, according to the literature, should increase material biocompatibility and biodegradability. Moreover, obtained PU foams were cost-effective comparing to PUs obtained from expensive isocyanates like 2,6-diisocyanatometyl caproate (LDI) or 1,4-diisocyanatobutane (BDI), but its nontoxic properties will be preserved. We conducted mechanical tests and dynamic mechanical and scanning electron microscopy analysis, and we observed interactions of obtained PUs with three media: canola oil, saline (0,9% NaCl water solution; pH = 5,5), and distilled water (pH = 7). Moreover, we examined hydrolytic degradation of obtained PU foams by incubating samples in phosphate buffered saline (pH = 7,4) for 36 weeks and we studied obtained PUs for their hemocompatibility by subjecting samples to human blood contact. Performed analyses let us conclude that only some of the obtained polyurethane foams are suitable for soft tissue engineering applications.

## 2. Materials and Methods

### 2.1. Materials

Polyol: poly(ethylene-butylene) adipate (Mw = 2000) (PEBA) (Purinova), 1,6-hexamethylene diisocyanate (HDI) (Aldrich), silicon (surfactant), 1,4-buthanediol (BDO) (POCH), 1-ethoxy-2-(2-hydroxyethoxy)ethanol (EHEE) (POCH), gelatin (the average size of grains is of the order of 3-4 *μ*m, gelatin type B1 180, Gelwe), water, 1, 4-diazabicyclo[2.2.2]octane (DABCO) (Aldrich), and potassium acetate (Aldrich).

### 2.2. Synthesis

Two series of PU foams (unmodified and gelatin-modified) from polyester PEBA, water, silicone, chain extender (BDO or EHEE), and HDI were obtained. Both unmodified and gelatin-modified PU foams were synthesized at four molar ratios of isocyanate groups to hydroxyl groups (NCO : OH= 0,8 : 1–1,1 : 1). Synthesis of polyurethane foams was carried out in two stages. First unmodified polyol mixtures, with different chain extenders (BDO or EHEE), were prepared in the glass reactor at 50°C for 4 h. Then heated at 50°C HDI was added to the polyol mixture. Compositions were subjected to intensive stirring for 30 seconds in homogenizer at a speed of 300 rev/min and then transferred into a mold and left at room temperature to complete the synthesis and to cure obtained PU foams. In the first series, we obtained eight samples of polyurethane foams, without gelatin, based on different types of chain extenders (BDO or EHEE) (Table 1). 

**Figure 1:**
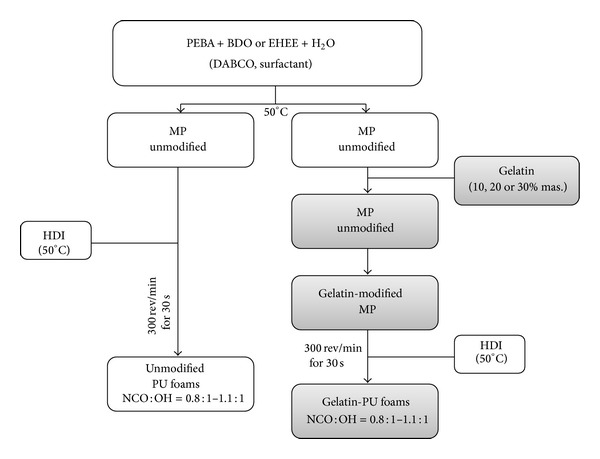
Synthesis of unmodified and gelatin-modified polyurethane foams.

Tensile strength evaluation of all unmodified PU foams and examination of their pores size and shape allowed us to choose some PU foams for further gelatin modification. Gelatin addition was as follows: the proper amount of powdered gelatin was added to unmodified polyol mixture (correspondingly 10%, 20%, or 30%), and then the mixture was mixed for 30 seconds in a homogenizer at a speed of 300 rev/min. Heated at 50°C, HDI was added. In this way, we received eight samples of gelatin-modified polyurethane foams based on different types of chain extenders (BDO or EHEE) and various amounts of gelatin (Figure 1).

## 3. Methods

**Figure 2:**
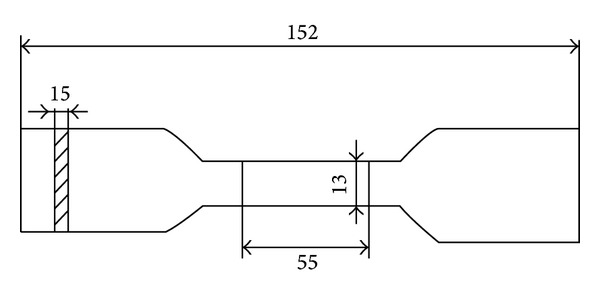
Dumbbell-shaped sample, with its dimensions according to PN-EN ISO: 1799 : 2009, used for the tensile strength test.

*Tensile strength* was performed by using the Zwick/Roell machine according to PN-EN ISO: 1799 : 2009. Dumbbell-shaped sample, of dimensions (measured in mm) shown in Figure 2, was fixed in the testing machine jaws. Then sample's dimensions were entered into the computer connected to the testing machine. The crosshead speed was of 500 mm/min ± 50 mm/min. The tensile strength results are the arithmetic mean of three measurements.

**Figure 3:**
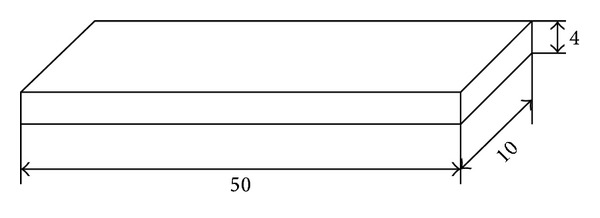
Beam-shaped sample, with its dimensions, used for the DMA analysis.

*Dynamic Mechanical Analysis (DMA)* was conducted on the Q800 DMA analyzer. Beam-shaped sample, of dimensions (measured in mm) presented in Figure 3, was placed in the testing machine. Then sample's dimensions were entered into the computer connected to the testing machine. The sample, placed in the holder, through the mandrel, was subjected to sinusoidal impact strength variable with a frequency of 1 and 10 Hz of constant amplitude. The sample was heated at a rate of 4°C/min from −100 to 50°C. The cooling medium in the chamber during the test liquid nitrogen was used. The DMTA results are the arithmetic mean of three measurements.

*Scanning Electron Microscope Analysis (SEM)* was used to analyze foams morphology. The samples before SEM analysis were coated with gold in turbopumped sputter coater (Quorum 150T E), and then they were viewed under Zeiss Scanning Electron Microscope EVO-40 at the magnification of 30 and 100 times. 

*Interactions with canola oil, saline, and distilled water* were carried out with samples in a size of 1 cm^2^. Before the test they were dried to the constant weight. Then they were transferred into a polyethylene container, filled with canola oil, saline, or distilled water. Samples were incubated in a drier at 37,0 ± 0,2°C. Canola oil sorption was measured after 24 h, and saline and distilled water sorption was taken after 3, 7, and 14 days of incubation [26]. Sorption percentage (*S*) was calculated according to formula (1) where *m*_*t*_ was the sample's weight after incubation (g) and the *m*_0_ was the sample's weight before the test (g). The sorption results are the arithmetic mean of three measurements as

(1) $$\begin{array}{c} S=\frac{\left(m_{t}-m_{0}\right)}{m_{0}}\cdot 100\%. \end{array}$$

*Polyurethane hydrolytic degradation* was measured with pseudodynamic method (buffer solution was changed when its value was reduced by 0,5 unit) [27]. Dried and weighted polyurethane samples of 1 cm^2^ were put into container with phosphoric buffer solution, which contained 0.02% of NaN_3_ (bacteriostatic substance). Then they were incubated at 37°C. Samples' weight changes were measured after 4, 12, 24, and 36 weeks of incubation (after rinsing samples with distilled water and drying at 60°C in vaccum, to a constant mass). The results are arithmetic mean of three measurements. pH of solution was controlled every two weeks. 

*Polyurethane hemocompatibility* was examined in Medical laboratory with analyzer SYSMEX XS-1000i. Samples of venous blood from two healthy women were used in this study [28]. Biologic material, directly after being taken, was put into test tube containing potassium acetate agent, which prevents blood clotting. Next step was obtaining reference parameters for blood morphology. After that were transferred to the test tube 8 cm^2^ of PU foams (sterilized before with argon gas plasma generated over H_2_O_2_) and 8 mL of taken blood. Samples made this way were incubated in blood for 15 minutes at room temperature. After this time polyurethane foams were removed. Blood, after 15 minutes of contact with polyurethane foams, was hematologically analyzed. 

## 4. Results

### 4.1. Unmodified PU Foams:Properties Evaluation

Unmodified PU foams were prepared according to the data presented in Table 1.

Then we measured the fundamental parameters for PUs, such as tensile strength, glass transition temperature, hard segments content, pore size, and growth time of the foams. All parameters, for unmodified PU foams, are presented in Table 2.

**Figure 4:**
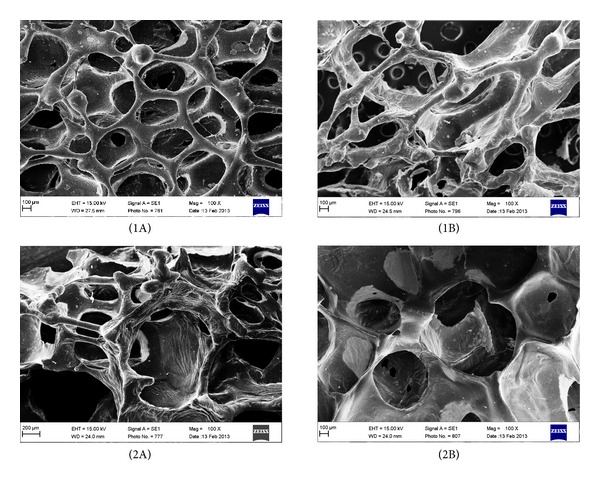
Normal (1A) and large (2A) pores of PUs in which chain extender was BDO and normal (1B) and large (2B) pores of PUs in which chain extender was EHEE. All images are viewed at 100x magnification.

Taking into account the data in Table 2, it is clear that PU-1/EHEE/G0 and PU-1/BDO/G0 were the most suitable for further modification with gelatin. The most important parameter the deciding parameter of samples choice was their morphology (pore size and shape)(Figure 4), as the rest parameters were comparable.

### 4.2. Gelatin-Modified PU Foams:Properties Evaluation

In Table 3 results for fundamental parameters of gelatin-modified PU foams are presented. The results will be discussed further with other data.

### 4.3. SEM Analysis

**Figure 5:**
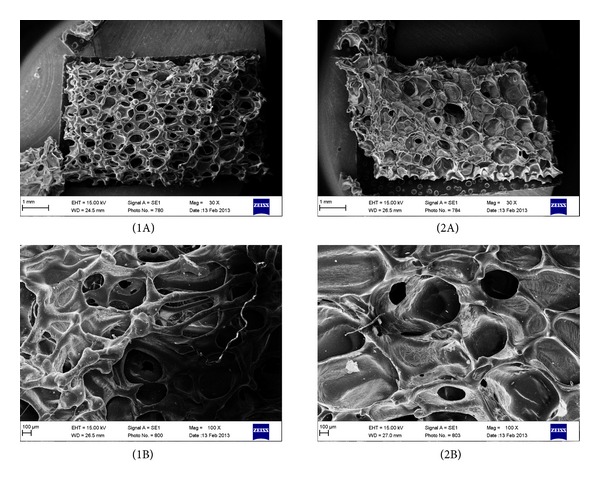
SEM of PU foams, in which chain extender was BDO, after gelatin modification with 20% of gelatin addition, viewed at ×30 (1A) and ×100 (1B) magnification, and 30% of gelatin addition, viewed at ×30 (2A) and ×100 (2B) magnification.

**Figure 6:**
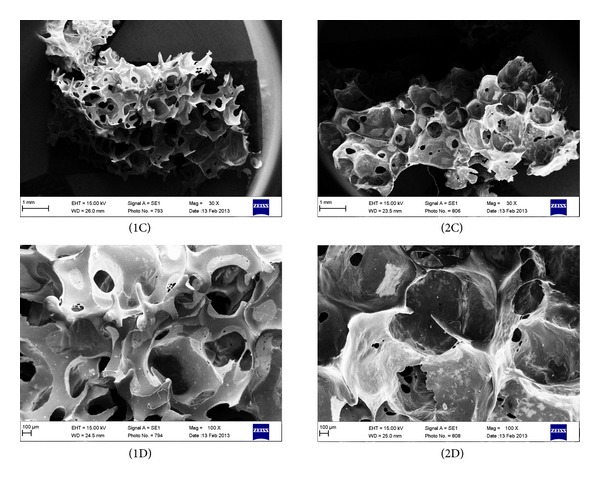
SEM of PU foams, in which chain extender was EHEE, after gelatin modification with 20% of gelatin addition, viewed at ×30 (1C) and ×100 (1D) magnification, and 30% of gelatin addition, viewed at ×30 (2C) and ×100 (2D) magnification.

Figures 5 and 6 present morphology for modified PU foams, in which chains were extended by BDO or EHEE and different amounts of gelatin were added (correspondingly 10%, 20%, and 30%).

Three-dimensional scaffolds should show a highly porous structure to allow a proper vascularisation of the implant, as well as the flow of nutrients and waste products. The porous structure should be highly interconnected specifically, with pore sizes in the range of hundreds of microns (100–1000 *μ*m), that is, comparable to the size of blood vessels and with pores present in amounts higher than 50–60 vol.% [29–31]. From Figure 5 it is clearly seen that PU foam (PU-1/BDO/G20), in which BDO was used as chain extender, possesses suitable porosity in shape and size (500–800 *μ*m) and gelatin granules are placed in the bulk wall of PU foams and they are in a size of 226–236 *μ*m. Foams PU-1/BDO/G10 and PU-1/BDO/G30 show much worse quality of the pores and moreover in PU-1/BDO/G30 pores are completely fulfilled with gelatin. PU foams, in which synthesis EHEE chain extender was used, have irregular pores morphology (Figure 6). 

### 4.4. Interactions with Canola Oil, Saline, and Distilled Water

These parameters were measured to get some point of view, how the porous PU scaffold will act with human body fluids after implantation. Canola oil, saline, and distilled water were chosen mainly because human body is built from fats, body fluids, and water. To our knowledge such studies have not been published so far for that type of polyurethanes. 

### 4.5. Canola Oil Sorption

Canola oil sorption was measured for both unmodified and modified PU foams, and the results are presented in Table 4. 

Unmodified PU foam, in which BDO chain extender was used (PU-1/BDO/G0) had higher value of canola oil sorption (14 ± 2%) than PU, in which EHEE chain extender was used (PU-1/EHEE/G0 = 4 ± 1%) (Table 4). After gelatin modification, we observed the enhancement of sorption level for PU foams obtained with both chain extenders. In this case, canola oil sorption value for foams modified with gelatin was much higher for those samples, in which BDO was used as chain extender (especially for PU foams having 10% or 20% of gelatin). In contrary, for PU-1/BDO/G30 sample canola oil sorption is lower than in the case of PU-1/BDO/G10 and PU-1/BDO/G20 samples. Gelatin-PU foams, having chains extended by EHEE, had lower sorption capability of canola oil. 

### 4.6. Saline Sorption and Distilled Water Sorption

In Table 5 data of saline and distilled water sorption for PU foams having two types of chain extenders and different amounts of gelatin are presented.

Table 5 shows that unmodified foam samples, in which BDO chain extender was used, undergo decomposition after 3 days of saline and distilled water incubation. In contrary, unmodified PU foams, in which EHEE was used as chain extender, stay stable after 14 days of incubation. Thus sorption value for gelatine modified PUs is much higher for samples, Thus sorption value for gelatine modified PUs is much higher for samples, in which synthesis was BDO used as chain extender than for those in which EHEE was used.

### 4.7. Hydrolytic Degradation of PU Foams

In Table 6 the mass loss data for gelatin modified PU foams after 4, 12, 24, and 36 weeks of incubation in phosphorous buffer solution are presented.

Foams without gelatin, regardless of the applied chain extender, undergo negligible degradation (Table 6). Mass loss of samples after 36 weeks of incubation, in which BDO was used as chain extender, was 3,5% while for EHEE it was 4,6%. Gelatin addition increases hydrolytic degradation capability. PU foams, in which BDO was chain extender, modified with gelatin in the amount of 10% (PU-1/BDO/G10) had mass loss from 54% to 64%. PU foams, in which EHEE was the chain extender (PU-1/EHEE)/G10), had mass loss from 45,3% to 72,3% after 36 weeks of examination time. Studies of hydrolytic degradation reported that, in case of saline and distilled water sorption, the mass changes (after longer time of incubation, for example, 36 weeks) are due to hydrolytic degradation of analyzed material. Ester groups and also presence of gelatin contribute to long-term material degradation. 

### 4.8. Hemocompatibility

Table 7 presents the reference values of blood parameters and its value for blood taken from healthy woman, which was later used for hemocyte compatibility tests.

**Figure 7:**
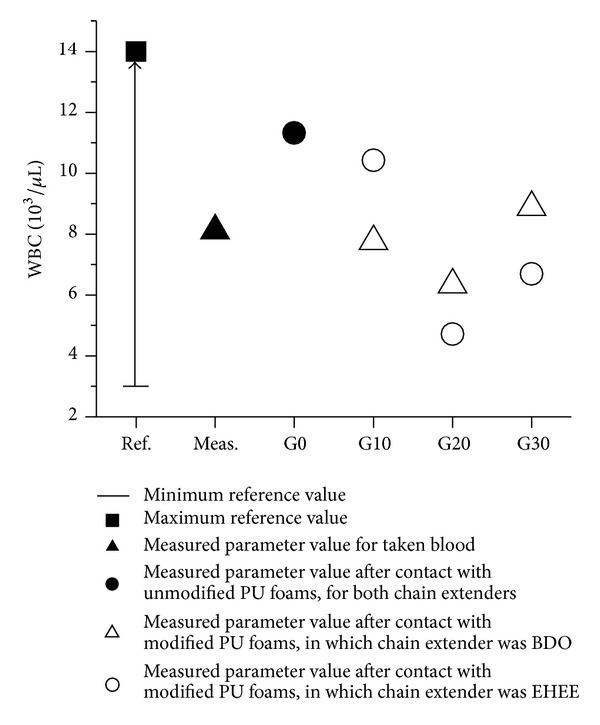
WBC value, for all investigated samples, which stays in the reference range.

**Figure 8:**
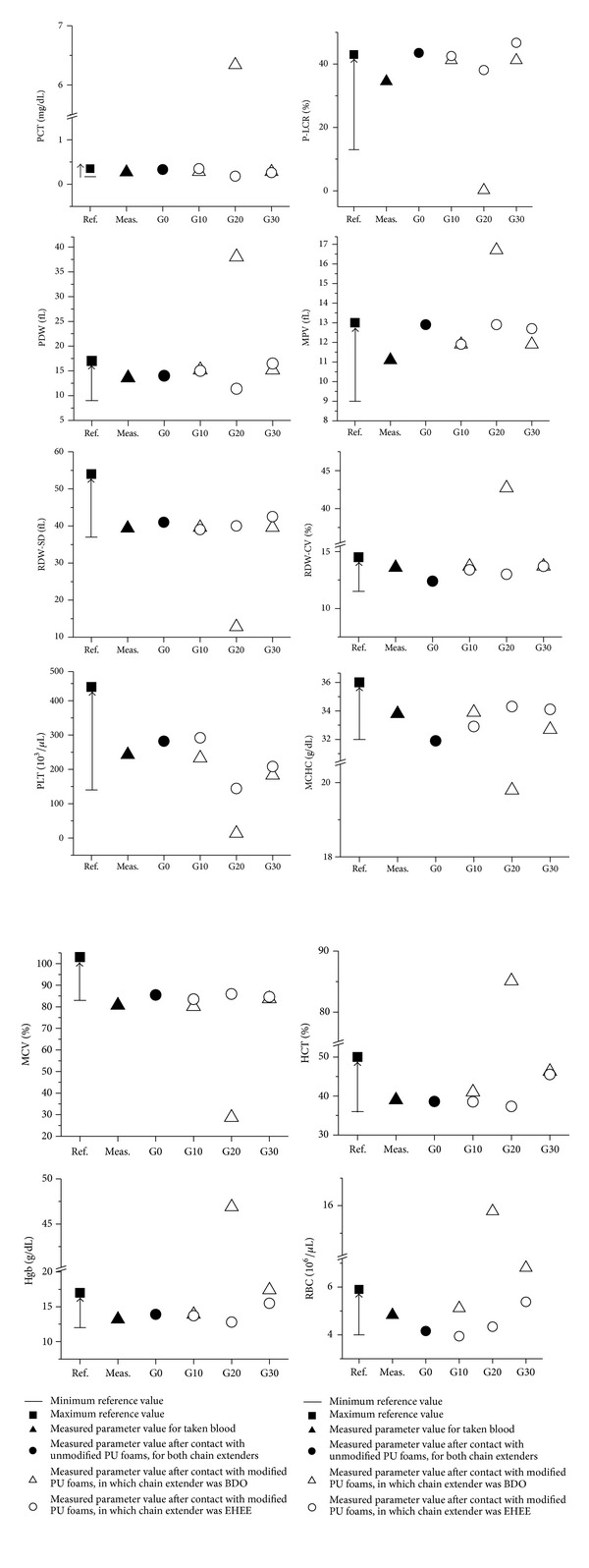
Blood parameters of all investigated samples, with values above reference range (some deviation from reference range was noticed, with a concentration on G20 PU samples, in which chain extender was BDO).

The WBC blood parameter was in the range of reference value for all samples investigated (Figure 7). Another blood parameters (Figure 8) were in the range of reference sample excluding PU-1/BDO/G20 sample.

## 5. Discussion

In our paper, we report the properties of unmodified and gelatin-modified PU foams in terms of medical application for soft tissue engineering. Unmodified PU samples differing in used chain extenders (BDO or EHEE) were prepared at four molar ratios of NCO:OH groups (0.8 : 1–1,1 : 1). All unmodified PU foams had tensile strength values in the range of 1.30 ± 0.47–3.21 ± 0.48 MP and that was comparable with the literature data for soft tissue engineering [32, 33]. As the tensile strength values remained within normal range for all obtained PU samples, the deciding parameter of our samples choice for further gelatin modification was their morphology (pores size and shape). We decided to select for further gelatin modification PU foams obtained at molar ratio of NCO : OH = 1 : 1. According to the literature data, they had the most suitable pore size and shape. Unmodified PU foams obtained at different molar ratios of NCO : OH than 1 : 1, had inadequate morphology. In case of EHEE chain extender, obtained PU foams had pores formed accidentally and of random sizes. On the other hand, in PU foams, where chain extender was BDO, pores were irregular in their size and shape and their interconnectivity was discursive. 

In case of gelatin-modified PU samples, the morphology was more regular in some cases and dependeds on chain extender type and amount of gelatin added. Gelatin-modified PU foam sample denoted as PU-1/BDO/G20, having 20% of gelatin and BDO used as chain extender, possessed desirable morphology for TE application. This sample has quasi-regular pores in shape and size (500–800 *μ*m). Otherwise in PU-1/BDO/G10 and PU-1/BDO/G30, gelatin partially closes the PU scaffold pores and acts as a filler and that kind of morphology is not suitable for TE. Careful analysis of SEM pictures shows that, in case of gelatin addition to PU, we observed films around foam pores, pores filled with gelatin, or gelatin granules attached to PU skeleton. Thin films around foam pores were present in PU with lower amount of gelatin (PU-1/BDO/G10). Pores filled with gelatin were present in PU-1/BDO/G30. While for PU-1/BDO/G20, there were present quasi-regular pores and granules of gelatin attached to PU skeleton. Thus the crucial role in good pore structure creation was the proper amount of gelatin added. During PU formation, in the presence of gelatin, the balance between the amount of gelatin and growth time of the foam as well as the foaming temperature should be preserved, as well as the other parameters like foaming temperature. We found that this balance is the most accurate in case of PU-1/BDO/G20 what gave us the best morphology for TE.

Irregular pores found for modified PU samples with EHEE used as chain extender can be explained by their hydrophobic structure (ether groups in their structure). Due to this fact, the system is more complicated than in case of hydrophilic chain extender. We preliminary tested compatibility properties of gelatin with both chain extenders and we observed that BDO chain extender has higher possibility to bind with gelatin (higher hydrophilic character and lower molecular weight in comparison to EHEE). 

Gelatin addition increases distilled water and saline sorption values and affects canola oil sorption regardless of the used chain extender. However, gelatin-PU foams, in which BDO chain extender was used, possessed increased sorption values than those in which chains were extended with EHEE. The highest sorption was observed for PU-1/BD/G20 sample, which had quasi-regular porosity, which allowed for medium retention in pores, which contributed to this sorption by its swelling ability at evaluated temperature (37°C). Moreover, in that sample gelatin acts as a binder that maintains PU structure. Additionally, gelatin functional groups such as carboxylic, amides, amines, and hydroxyls may partially react with NCO groups which keeps together the foam construction for longer time than that for unmodified foams. Otherwise, lower sorption values for PU foams, in which EHEE was used as chain extender, may be explained by hydrophobic nature of ether linkages occurring in EHEE chain structure and also by their probability to form hydrogen bonds. Unmodified polyurethanes undergo slower hydrolytic degradation, but their mass loss is neat. The PU modified with gelatin undergoes quicker degradation and mass loss is around 50%. Hydrolytic degradation studies demonstrated unequivocally that, with the increase of gelatin addition to the Pus, it also increases their mass loss. That may be explained by gelatin degradation, over time, which causes increase of polyurethane porosity which contributes to its degradation. Blood parameters, after contact with almost all samples investigated (with the exception of PU-1/BDO/G-20), did not change. Thus gelatin-modified PU foams are hemocompatible. In Table 8 we summarized all the examined parameters to choose the best sample for soft tissue scaffolding. Taking into account all parameters, the most suitable for TE is PU-1/BDO/G20.

## 6. Conclusions

We obtained a series of novel unmodified PU foams in two-step polymerization process from 1,6-hexamethylene diisocyanate (HDI), poly(ethylene-butylene adipate) (PEBA), and 1,4-butanediol (BDO) or 1-ethoxy-2-(2-hydroxyethoxy)ethanol (EHEE) used as chain extenders. After examination of *T*_SB_, growth time, *T*_g_, and pore size/shape evaluation of unmodified PU foams, we selected for further gelatin modification PU samples prepared at ratio of NCO/OH=1 : 1. Both unmodified and modified PU foams were examined towards their interactions with canola oil, saline, and distilled water, hydrolytic degradation and hemocompatibility with human blood. Properties evaluation showed that PU foams, in which BDO chain extender was used, possess higher canola oil, saline, and distilled water sorption capacity than those obtained from EHEE chain extender. Hemocompatibility studies demonstrated that modified PU foams, in which chain extender was EHEE, possess better interactions with blood than those obtained from BDO. SEM analysis showed that sample containing 20% of gelatin (PU-1/BDO/G20) has quasi-regular porosity in pore shape and size (500–800 *μ*m) and gelatin acts as a filler and its granules in a size of 226–236 *μ*m are visible in bulk wall of PU foams. Polyurethane sample, in which chain extender was BDO and the amount of gelatin was 20% appeared to be the most suitable for biomedical application such as soft tissue scaffolds.

## Figures and Tables

**Table 1:** The symbols and the molar ratio of substrates used in the synthesis of unmodified PU foams.

Type	Symbol	Polyester PEBA	Chain extender BDO	Chain extender EHEE	Water	Catalyst DABCO	Isocyanate HDI
Series I	PU-0.8/BDO/G0*	0.582	0.116	—	0.017	0.008	0.278
	PU-0.9/BDO/G0	0.560	0.112	—	0.017	0.007	0.303
	PU-1/BDO/G0	0.544	0.109	—	0.016	0.007	0.323
	PU-1.1/BDO/G0	0.525	0.105	—	0.016	0.007	0.347

Series II	PU-0.8/EHEE/G0	0.539	—	0.178	0.016	0.007	0.260
	PU-0.9/EHEE/G0	0.522	—	0.172	0.016	0.007	0.283
	PU-1/EHEE/G0	0.507	—	0.167	0.015	0.007	0.303
	PU-1.1/EHEE/G0	0.490	—	0.1618	0.015	0.007	0.327

*PU: polyurethane; 0.8 molar ratio of NCO : OH (0.8 : 1); BDO or EHEE: type of chain extender.

**Table 2:** Characteristics of unmodified PU foams.

Symbol	*T*_SB_ (Mpa)	Growth time (s)	*T*_*g*_ (°C)	Hard segments content (%)	Pore size
PU-1.1/BDO/G0	1.5 ± 1	1.30 ± 0.47	−30	45	Normal^1A^
PU-1/BDO/G0	1.2 ± 1	1.25 ± 0.49	−29	43	

PU-0.8/BDO/G0	1.3 ± 1	2.35 ± 0.46	−31	39	Large^2A^
PU-0.9/BDO/G0	1.4 ± 1	2.30 ± 0.45	−30	41	

PU-1.1/EHEE/G0	2.1 ± 1	1.31 ± 0.48	−30	40	Normal^1B^
PU-1/EHEE/G0	1.9 ± 1	1.30 ± 0.45	−28	39	

PU-0.9/EHEE/G0	1.45 ± 1	2.43 ± 0.46	−30	37	Large^2B^
PU-0.8/EHEE/G0	1.2 ± 1	3.21 ± 0.48	−29	35	

**Table 3:** Characteristics of gelatin-modified PU foams.

Symbol	*T*_SB_ (MPa)	Growth time (s)	*T*_*g*_ (°C)	Hard segments content (%)
PU-1/BDO/G10*	1.2 ± 1	1.45 ± 0.45	−31	58
PU-1/BDO/G20	1.5 ± 1	1.51 ± 0.47	−32	54
PU-1/BDO/G30	1.6 ± 1	2.01 ± 0.49	−31	49

PU-1/EHEE/G10	1.44 ± 1	1.43 ± 0.46	−30	48
PU-1/EHEE/G20	2.1 ± 1	2.01 ± 0.48	−31	44
PU-1/EHEE/G30	2.2 ± 1	2.31 ± 0.47	−32	41

*G10: 10% of gelatin addend.

**Table 4:** Canola oil sorption of PU foams prepared from different chain extenders and various amounts of gelatin.

Chain extender BDO		Chain extender EHEE	
PU symbol	Sorption (%)	Sorption (%)	PU symbol
PU-1/BDO/G0	14 ± 2	4 ± 1	PU-1/EHEE/G0
PU-1/BDO/G10	18 ± 3	7 ± 1	PU-1/EHEE/G10
PU-1/BDO/G20	19 ± 2	6 ± 2	PU-1/EHEE/G20
PU-1/BDO/G30	9 ± 1	5 ± 1	PU-1/EHEE/G30

**Table 5:** Saline and distilled water sorption of PU foams prepared with different chain extenders and various amounts of gelatin.

Symbol	Sorption (%)					
	3rd day		7th day		14th day	
	Saline	Distilled water	Saline	Distilled water	Saline	Distilled water
BDO chain extender						
PU-1/BDO/G0	17 ± 4	Degradation	Degradation	Degradation	Degradation	Degradation
PU-1/BDO/G10	27 ± 1	16 ± 1	21 ± 2	10 ± 4	14 ± 2	8 ± 2
PU-1/BDO/G20	33 ± 6	26 ± 6	24 ± 2	23 ± 2	15 ± 7	19 ± 4
PU-1/BDO/G30	27 ± 1	20 ± 3	17 ± 1	16 ± 2	9 ± 1	13 ± 3

EHEE chain extender						
PU-1/EHEE/G0	4 ± 1	6 ± 2	2 ± 1	3 ± 2	1 ± 1	2 ± 1
PU-1/EHEE/G10	7 ± 2	9 ± 1	3 ± 2	7 ± 1	2 ± 1	4 ± 1
PU-1/EHEE/G20	6 ± 2	13 ± 1	5 ± 1	9 ± 1	3 ± 1	6 ± 1
PU-1/EHEE/G30	4 ± 1	10 ± 1	2 ± 1	7 ± 2	1.6 ± 1	4 ± 2

**Table 6:** Mass loss data for PU foams after hydrolytic degradation.

Symbol	Mass change (%)			
	Weeks			
	4	12	24	36
PU-1/BDO/G0	3.2	1.7	−1.5	−3.5
PU-1/BDO/G10	1.1	−13.4	−21.3	−54.3
PU-1/BDO/G20	2.1	−9.2	−17.3	−57.2
PU-1/BDO/G30	1.0	−11.2	−23.4	−68.3

PU-1/EHEE/G0	4.5	3.1	−2.3	−4.6
PU-1/EHEE/G10	−1.1	−10.2	−32.3	−45.3
PU-1/EHEE/G20	−2.4	−13.7	−39.4	−54.3
PU-1/EHEE/G30	−3.8	−18.7	−44.3	−72.3

**Table 7:** Reference values of blood parameters and its value for blood taken to the studies of PU foams.

Blood parameters*	Unit	Reference value	Measured parameters of taken blood
WBC	10^3^/*μ*L	3.00–14.00	8.11
RBC	10^6^/*μ*L	4.00–5.90	4.83
Hgb/Hb	g/dL	12.00–17.00	13.2
Hct	%	36.00–50.00	39.0
MCV	%	83.00–103.00	80.7
MCHC	g/dL	32.00–36.00	33.8
PLT	10^3^/*μ*L	140–440	243
RDW-CV	%	11.50–14.50	13.6
RDW-SD	fL	37.00–54.00	39.4
MPV	fL	9.00–13.00	11.1
PDW	fL	9.00–17.00	13.6
P-LCR	%	13.00–43.00	34.5
PCT	mg/dL	0.17–0.35	0.27

*WBC: white blood cells—leucocytes; RBC: red blood cells—erythrocytes; Hgb/Hb: hemoglobin; Hct: hematocrit; MCV: mean corpuscular volume; MCHC: mean concentration of hemoglobin in blood cells; RDW: distribution volume of red blood cells; PLT: platelet amount—thrombocytes; PCT: percentage of platelets in whole blood volume; PDW: indicator of platelet volume distribution; MPV: mean platelet volume; GPCCR: parameter specifies the number of large plates.

**Table 8:** The summary of unmodified and modified PUs interactions with media, hydrolytic degradation, morphology, and hemocompatibility.

Symbol	Morphology (SEM)	Interactions with media	Hydrolytic degradation	Hemocompatybility
Before gelatin modification				
PU-1,1/BDO/G0	±	−	−	±
PU-1,1/EHEE/G0	−	−	−	±

After gelatin modification				
PU-1/BDO/G10	±	±	±	±
**PU-1/BDO/G20**	**+**	**+**	**±**	**±**
PU-1/BDO/G30	−	−	−	−
PU-1/EHEE/G10	−	−	+	+
PU-1/EHEE/G20	−	−	+	+
PU-1/EHEE/G30	−	−	+	+

Symbols: +: properties; ±: intermediate properties; −: poor properties.
